# Junctional ectopic tachycardia following tetralogy of fallot repair in children under 2 years

**DOI:** 10.1186/s13019-018-0749-y

**Published:** 2018-06-05

**Authors:** Mohamed Fouad Ismail, Amr A. Arafat, Tamer E. Hamouda, Amira Esmat El Tantawy, Azzahra Edrees, Abdulbadee Bogis, Nashwa Badawy, Alaa B. Mahmoud, Ahmed Farid Elmahrouk, Ahmed A. Jamjoom

**Affiliations:** 1 Cardiothoracic Surgery Department, King Faisal Specialist Hospital and Research Center, MBC J-16, P.O Box: 40047, Jeddah, 21499 Saudi Arabia; 2 Cardio-thoracic Surgery Department, Mansoura University, Mansoura, Egypt; 3 Cardiothoracic Surgery Department, Tanta University, Tanta, Egypt; 4 Cardio-thoracic Surgery Department, Benha University, Benha, Egypt; 5 The Department of Pediatrics, Faculty of Medicine Cairo University, Cairo, Egypt

**Keywords:** Congenital heart disease, Arrhythmia, Junctional ectopic tachycardia; tetralogy of Fallot

## Abstract

**Background:**

Junctional ectopic tachycardia is a serious arrhythmia that frequently occurs after tetralogy of Fallot repair. Arrhythmia prophylaxis is not feasible for all pediatric cardiac surgery patients and identification of high risk patients is required. The objectives of this study were to characterize patients with JET, identify its predictors and subsequent complications and the effect of various treatment strategies on the outcomes in selected TOF patients undergoing total repair before 2 years of age.

**Methods:**

From 2003 to 2017, 609 patients had Tetralogy of Fallot repair, 322 were included in our study. We excluded patients above 2 years and patients with preoperative arrhythmia. 29.8% of the patients (*n* = 96) had postoperative JET.

**Results:**

JET patients were younger and had higher preoperative heart rate. Independent predictors of JET were younger age, higher preoperative heart rate, cyanotic spells, non-use of B-blockers and low Mg and Ca (*p* = 0.011, 0.018, 0.024, 0.001, 0.004 and 0.001; respectively). JET didn’t affect the duration of mechanical ventilation nor hospital stay (*p* = 0.12 and 0.2 respectively) but prolonged the ICU stay (*p* = 0.011). JET resolved in 39.5% (*n* = 38) of patients responding to conventional measures. Amiodarone was used in 31.25% (*n* = 30) of patients and its use was associated with longer ICU stay (*p* = 0.017). Ventricular pacing was required in 4 patients (5.2%). Median duration of JET was 30.5 h and 5 patients had recurrent JET episode. Timing of JET onset didn’t affect ICU (*p* = 0.43) or hospital stay (*p* = 0.14) however, long duration of JET increased ICU and hospital stay (*p* = 0.02 and 0.009; respectively).

**Conclusion:**

JET increases ICU stay after TOF repair. Preoperative B-blockers significantly reduced JET. Patients with preoperative risk factors could benefit from preoperative arrhythmia prophylaxis and aggressive management of postoperative electrolyte disturbance is essential.

## Background

Postoperative junctional ectopic tachycardia (JET) is a potential life-threatening arrhythmia occurring after congenital cardiac surgery. [1] The incidence of JET following congenital cardiac surgery varies widely in literature which can be attributed to the different diagnostic criteria and the great variability in the patients’ characteristics among the published studies. [2] Incidence of JET is higher when the intervention is close to the atrioventricular node and bundle of Hiss as in tetralogy of Fallot (TOF) and complete atrioventricular canal (AVC) repair. [1, 3] Several treatment strategies ranging from pharmacologic agents to atrial cardiac pacing are used sequentially to lower the ventricular rate and re-establish atrioventricular synchrony. [4–6] Recently, the effect of several pharmacologic agents on reducing postoperative JET was evaluated. [7–9]

Generalization of preoperative prophylaxis for arrhythmia in all patients undergoing surgery for congenital heart defects is non-practical and preoperative identification of high risk patients is essential. Although many studies were concerned with postoperative arrhythmia, little have focused on JET following total TOF repair in infants. [1, 10] The objectives of this study were to characterize patients with JET, identify its predictors and subsequent complications and the effect of various treatment strategies on the outcomes in selected TOF patients undergoing total repair before 2 years of age.

## Methods

### Study population

This is a retrospective cohort study performed at King Faisal Specialized Hospital and Research Centre in Jeddah, Saudi Arabia. A total of 609 patients underwent surgical TOF repair between January 2003 and December 2017. We excluded patients older than 2 years (*n* = 231) and patients with rhythm disturbances or heart block preoperative (*n* = 16). Moreover; patients with serious postoperative arrhythmia other than JET (*n* = 17) and patients with missing postoperative JET diagnosis criteria from the medical records (*n* = 23) were excluded. A total of 322 patients were included in the study. Approval of the institutional review board was obtained before data collection and the need for patients’ consents was waived due to the retrospective nature of the study.

### Operative technique

Surgical repair was performed by consultant level cardiac surgeons through median sternotomy. Bicaval cannulation was performed and cardioplegic arrest was done by antegrade cold crystalloid cardioplegia. Median temperature of the perfusate was 32 °C. Right atrial incision was performed in 98% of the patients for resection of the RVOT obstructing bundles and closure of the ventricular septal defect and trans-annular patch (TAP) was used in 64% of the patients. Patients who required TAP were mainly operated upon during the early study period and our current strategy is to avoid TAP and preserve the pulmonary valve when possible. Pulmonary valvotomy was performed in 71 patients (22.5%) and resection of the right ventricular outflow tract (RVOT) obstructing bundles was done in 94.7% (*n* = 305). Concomitant tricuspid valve repair was performed in 55 patients (17.4%).

### Data collection

Patients’ medical charts were retrospectively reviewed to collect preoperative variables including patients age at the time of surgery, gender, weight (Kg), body surface area (BSA) (m2), preoperative B-blockers administration, associated cardiac anomalies and previous palliative surgical procedures as modified Blalock-Tausig Shunt. Preoperative 12 leads electrocardiogram were reviewed in all patients to identify the preoperative rhythm and PR interval, QRS duration were calculated. Preoperative heart rate was reported from the anesthesia records after sedating the patients. Intraoperative variables include cardiopulmonary bypass (CPB) time (minutes), aortic cross clamp time (minutes), temperature of the perfusate, use of transannular patch (TAP) and resection of right RVOT obstructing muscle bundle and the use of right atrial or right ventricular approach.

### Postoperative data

We included all patients who had JET whatever its duration and the onset of JET whether intraoperatively or postoperatively in the intensive care unit (ICU) was determined. The criteria used for JET diagnosis were i) Heart rate > 175 Bpm ii) Absent P wave from lead II of EKG iii) Narrow QRS complexes iv) Ventricular rate faster than atrial rate with AV dissociation.

Protocol of JET management included discontinuation of unnecessary inotropes, infusion of IV fluids boluses for hypovolemic patients with low central venous pressure, cooling (reducing temperature to 36–36.5 °C), and sedation. At the same time postoperative electrolyte imbalance was aggressively corrected with K, Ca, and magnesium. Our Intensive care protocol is to maintain serum K > 4.1 mmol/L, ionized Ca > 1.1 mmol/L and serum Mg > 1.1 mg/dL. During early study period, digoxin was administered at 5 μcg /kg/dose IV once to control the ventricular rate. Amiodarone was administered as a bolus with 5 mg / kg IV over one hour and if JET persisted, further infusion at 5 μcg /kg/min was given till sinus rhythm was established or the heart rate slowed to an acceptable rate with stable hemodynamic. Our policy for possible postoperative pacing is to insert ventricular pacing wire unless the patient showed heart block immediately on recovery from CPB, in this case we insert both atrial and ventricular pacing wires. Dopamine, dobutamine, milrinone, epinephrine administration was reported, and we calculated the inotropic score at the time of onset of arrhythmia using the following formula: {(dopamine + dobutamine) × 1} + (milrinone X 20) + {(epinephrine + norepinephrine) × 100}.

### Study endpoints

Preoperative, operative and postoperative variables were used to predict the occurrence of JET. The impact of JET on duration of mechanical ventilation (hours), ICU stay (days) and total duration of hospital stay (days) were analyzed.

### Statistical analysis

#### Data presentation

Continuous variables were presented as mean ± Standard deviation and categorical variables as number and percent.

#### Data analysis

Mann-Whitney test was used to compare continuous variables and for categorical variables Chi2 was used or Fisher Exact test if the frequency is less than 5. Multiple imputation was used to handle the missing variables. Multivariable logistic regression models were used to identify the predictors of postoperative JET. Three models were constructed from the preoperative, operative and postoperative variables respectively and the variables in each model didn’t exceed 9 variables to power the multivariable analysis. Odds ratio, *p*-value and 95% confidence interval were reported. The effect of JET on the postoperative outcomes was assessed using propensity score analysis. The probability of having JET was calculated using multivariable logistic regression model after adjustment of the measured preoperative confounders. Propensity score was used in the adjusted model to predict the effect of JET on the postoperative outcomes. To fulfil the linearity assumptions of the model, non-normally distributed continuous variables were rescaled by, inverse squared and inverse transformations. *P*-value lesser than 0.05 was considered significant. All statistical analyses were performed using STATA 14 software (Statacorp, Texas- USA).

## Results

A total of 322 patients had tetralogy of Fallot repair during the study period and met the inclusion criteria. Patients’ age at time of operation ranged from 2 to 23 months and male to female ratio was 1.6: 1. JET occurred in 96 patients (29.9%) and patients were classified into JET and non-JET groups.

### Patients’ characteristics

Patients who had postoperative JET were significantly younger (*p* = 0.01) and had higher preoperative heart rate (*p* = 0.03). Associated lesions were Atrial Septal Defect (*n* = 6), complete AVC (*n* = 3), persistent Left Superior Vena Cava (*n* = 1), Patent Ductus Arteriosus (*n* = 7), Multiple Aorto- Pulmonary Collateral Arteries (*n* = 2), peripheral pulmonary artery stenosis (*n* = 3), pulmonary atresia (*n* = 5) and 12 patients had prior modified Blalock Taussig shunt. There was no statistically significant difference in associated lesion between patients with and without postoperative JET (*p* = 0.8). Fifteen patients in JET group had preoperative B-blockers (19.5%) versus 62 patients (80.5%) in Non-JET group (*p* = 0.015). (Table 1).

**Table 1:** Comparison of the preoperative and operative data between JET and non-JET patients

Variable	JET group (*n* = 96)	Non-JET (*n* = 226)	P
Male (n)	56 (58.3%)	144 (63.7%)	0.36
Age (months)	7.5 ± 3	8.35 ± 2.99	0.01
Weight (Kg)	6.95 ± 1.8	7.37 ± 2.1	0.11
BSA (m2)	0.36 ± 0.07	0.37 ± 0.6	0.42
Oxygen saturation (%)	82.4 ± 7.7	82.8 ± 8	0.14
Cyanotic spells (n)	20 (21.7%)	42 (19%)	0.58
EKG			
Heart Rate (b/m)	103.7 ± 13.5	99.97 ± 12.97	0.03
P duration	0.06 ± 0.01	0.06 ± 0.01	0.7
P-R interval	0.13 ± 0.02	0.13 ± 0.03	0.7
QRS	0.06 ± 0.02	0.06 ± 0.01	0.5
Pulmonary annulus (mm)	6.36 ± 2	6.47 ± 2.1	0.57
RVOT gradient (mmHg)	70.8 ± 14.6	71.5 ± 15.1	0.61
Cross-Clamp time (min)	74.4 ± 2.6.5	75.3 ± 27.1	0.77
Cardiopulmonary bypass (min)	102.1 ± 31.1	101.3 ± 32	0.7
Temperature (Celsius)	32 ± 1.5	31.7 ± 1.5	0.12
Tricuspid valve repair (n)	13 (13.5%)	42 (19%)	0.24
Pulmonary valvotomy (n)	25 (26.6%)	47 (20.7%)	0.25
RA approach (n)	93 (96.9%)	223 (98.7%)	0.28
RVOT resection (n)	91 (94.8%)	214 (94.7%)	0.97
Trans-annular patch (n)	66 (68.75%)	141 (62.39%)	0.27

Continuous variables are presented as mean ± SD and categorical variables as number (%)

*RA* right atrial, *RVOT* right ventricular outflow tract

Operative variables are comparable between both groups with no statistically significant difference in the measured variables. Table 1 shows the comparison of the preoperative and operative variables. Postoperative inotropes administered before the onset of JET were compared between groups and showed no significant difference in patients who received dopamine, dobutamine, epinephrine and milrinone between JET and non-JET patients. Levels of serum potassium, calcium and magnesium before the onset of JET were compared and showed significantly lower magnesium level in patients who developed JET (*p* = 0.001). (Table 2).

**Table 2:** Comparison of the inotropes and serum electrolytes in JET and non-JET groups

Variable	JET group (*n* = 96)	Non-JET (*n* = 226)	P
Inotropes			
Dopamine (n)	22 (23.4%)	65 (29.28%)	0.28
Dobutamine (n)	47 (49.5%)	91 (41%)	0.16
Epinephrine (n)	34 (36.6%)	85 (37.8%)	0.84
Milrinone (n)	47 (50%)	127 (58.3%)	0.18
Inotrope score	12.3 ± 14	9.2 ± 6	0.17
Electrolytes			
PH	7.36 ± 0.066	7.37 ± 0.06	0.16
Potassium (mmol/L)	3.98 ± 0.45	4.07 ± 0.56	0.17
Magnesium (mEq/L)	1.01 ± 0.39	1.14 ± 0.43	0.001
Calcium (mmol/L)	2.32 ± 0.32	2.24 ± 0.35	0.08

Continuous variables are presented as mean ± SD and categorical variables as number (%)

### Factors associated with JET

By multivariable analysis of the measured preoperative variables, younger age, cyanotic spells, non-use of B-blockers and higher preoperative heart rate predicted the postoperative JET (*p* = 0.011, 0.024, 0.001 and 0.018; respectively). By constructing a separate model for the operative variables, none of the measured operative variables predicted the postoperative JET. In the postoperative variables model; low magnesium and calcium independently predicted the occurrence of JET (*p* = 0.004 and 0.001; respectively). (Table 3).

**Table 3:** Predictors of postoperative JET

Variable	OR	P	95% CI
Pre-operative			
Age	0.86	0.011	0.76–0.97
Cyanotic spells	2.9	0.024	1.15–7.41
B-blockers	0.2	0.001	0.08–0.51
Preoperative HR	1.02	0.018	1.004–1.04
Post-operative			
Mg	0.37	0.004	0.19–0.7
Ca	0.4	0.001	0.23–0.7

*OR* odds ratio, *CI* confidence interval

### Effect of JET

Ventilation time didn’t differ significantly between JET patients (23.4 ± 24.7 h) vs non-JET patients (19.5 ± 11.6 h, *p* = 0.11). Patients with JET had longer ICU stay (6.1 ± 5.8 vs 5 ± 1.9 days, *p* = 0.01) and longer hospital stay (15.1 ± 11.2 vs 13.4 ± 6.5 days, *p* = 0.04). Propensity score matching was used to estimate the effect of JET on the duration of ventilation, ICU and hospital stay after adjustment of the measured preoperative variables. JET didn’t affect the duration of mechanical ventilation nor hospital stay (*p* = 0.12 and 0.2 respectively) but significantly prolonged ICU stay (*p* = 0.011). (Table 4) Hospital mortality occurred in 10 patients (3.11%), 4 patients with JET (4.1%) versus 6 patients without JET (2.65%) (*p* = 0.49). (Table 4).

**Table 4:** Effect of JET on duration of mechanical ventilation, ICU and hospital stay

Variable	Coefficient	P	95% CI
Ventilation time^a^	−1.5	0.123	−0.036–0.004
ICU stay^b^	−0.3	0.011	−0.057–-0.007
Hospital stay^a^	− 0.009	0.2	−.025–.007

^a^inverse squared

^b^inverse

### Course of JET

JET was diagnosed post cardiopulmonary bypass and inside the operation room in 30 patients (30.25%) and in the ICU in 66 patients (68.75%). Subgroup analysis was done to identify the difference between patients who had JET intraoperatively and those who had JET in the ICU. No difference between both groups as regard age (*p* = 0.17), gender (*p* = 0.82), body surface area (*p* = 0.89), preoperative heart rate (*p* = 0.86), preoperative oxygen saturation (*p* = 0.37), cyanotic spells (*p* = 0.86), B blockers (*p* = 0.65), CPB time (*p* = 0.11) and ischemic time (*p* = 0.24). After adjustment of the preoperative variables, no difference was found in duration of JET (*p* = 0.91), ICU stay (*p* = 0.43), ventilation time (*p* = 0.52) and hospital stay (0.14) between both groups.

JET resolved in 39.5% (*n* = 38) of patients responding to conventional measures. At the same time, postoperative electrolyte imbalance was aggressively corrected with K, Ca, and Mg. Fifty-nine patients (61.5%) received digoxin and digoxin administration was not significantly associated with the duration of mechanical ventilation (*p* = 0.77), ICU stay (*p* = 0.43) nor hospital stay (*p* = 0.98) in patients with JET. Amiodarone was used in 31.25% (*n* = 30) of patients and no relation was found between the use of amiodarone and the duration of mechanical ventilation (*p* = 0.07) nor hospital stay (*p* = 0.35). However, amiodarone use was significantly associated with longer ICU stay (*p* = 0.017). Betablockers were used in 58 patients (64.2%) with no significant association with the duration of mechanical ventilation (*p* = 0.8), ICU (*p* = 0.37) or hospital stay (*p* = 0.07). Ventricular pacing was required in 4 patients (4.16%) because of progression into heart block. Five patients (5.2%) had a second episode of JET, 3 of them were males. Their median age was 7 months (ranged from 6 to 15 months) and median oxygen saturation was 80% (ranged from 79 to 85%). None of these patients had preoperative B-blockers, 3 of them had TAP and JET occurred intraoperatively in 3 patients. Full recovery occurred in 4 patients and 1 patients progressed to complete heart block and required permanent pace maker.

The median duration of JET was 30.5 h and ranged from 3 to 96 h. Longer duration of postoperative JET “i.e. above the median value” was significantly associated with lower preoperative oxygen saturation (p = 0.01). After adjustment of the pre- JET variables, longer duration of JET was significantly associated with prolonged ICU (*p* = 0.02) and hospital stay (*p* = 0.009) but had no significant association with the duration of mechanical ventilation (*p* = 0.21) compared with patients with short JET duration.

## Discussion

Prophylaxis of arrhythmia after pediatric cardiac surgery became the focus of many trials recently. [7, 8, 11, 12] Generalization of prophylaxis in all patients undergoing surgery for congenital heart disease is difficult and may increase the hazard of surgery. In literature, various factors were found associated with the genesis of JET after surgical repair of congenital heart disease. Most of these studies [1, 10, 13–15] were performed in a wide variety of congenital heart defects. Including all types of congenital heart defects in risk models to predict postoperative JET underpowers the results as the incidence of JET varies widely in different congenital heart diseases and procedures. Based on several reports, JET is commonly associated with TOF [1, 10] therefore we included in our series TOF patients who underwent corrective surgery before 2 years of age. In our series the incidence of JET was 29.8% and this high incidence of JET can be explained by our strict selection criteria of those high-risk patients. The incidence of JET following TOF repair varied in the published series and ranged from 4 to 37%. [8, 16–18] The great variability in JET incidence could be attributed to the diagnostic criteria used and the wide variability in patients’ characteristics especially age. [2] Another explanation for this variability is the low patients number, it is remarkable that lower incidence of JET post TOF repair was documented with less number of TOF patients included in the study. Moreover, we included all patients who expressed JET whether were hemodynamically stable or not. In our study; younger age was significantly associated with increased risk of postoperative JET and this finding is consistent with other studies [14]. Younger patients are generally sicker and smaller hearts are more prone to damage by surgical technique and retraction. Increased preoperative heart rate predicted postoperative JET. Normal range of heart rate depends on patients’ age and the definition of tachycardia is not consistent. In order to have a standardized condition for all patients, we reported the operation room heart rate after the patients were properly sedated. This also could explain the insignificant findings of the preoperative ECG measurements as usually they are not taken under standardized conditions and patients conditions during ECG recording significantly affected these ECG intervals.

Preoperative B-blockers were associated with significant reduction of postoperative JET in our patients which is consistent with a previous report. [19] Further randomized studies are required to evaluate Bblockers as a preoperative prophylaxis against JET in those high-risk patients.

Trans-atrial approach is our standardized approach for closure of ventricular septal defect and relief of RVOT obstruction and 98% of our patients were operated through RA. Although RA approach in a study of 343 patients independently predicted postoperative JET in the total patient series, no correlation was made with their 114 subsets of TOF patients. [20] Our surgical strategies have been shifted from the use of TAP to preservation of the pulmonary valve if possible as it has a proved efficacy in reducing the incidence of postoperative pulmonary regurgitation. [21] Nevertheless, TAP was not associated with JET in our series. Some authors documented a significant association between JET and prolonged ischemic and cardiopulmonary bypass time and higher bypass temperature [17, 22–24]. In our study, none of the operative variables predicted the postoperative JET. Cardiopulmonary bypass time affected JET in studies which included all congenital heart disease patients. In those patients, CPB time differed significantly between different lesions due to the different surgical procedure specific to each lesion Inotropes didn’t predict JET in our patients. In contrary to this, dopamine or inotropic score were risk factors for JET in other series. [14, 17] Revision of electrolyte profiles in the sample just prior to JET occurrence proved that JET group had statistically significant lower serum magnesium and Calcium. Serum magnesium carries a lot of debate among authors, some documented its significance as a risk factor or as prophylactic therapy for JET, [25–27] others documented that magnesium and calcium levels were not significantly different between the two groups. [22]

Our experience in management of JET is to imply conventional strategies as correction of reduced intravascular volume, reduce body temperature, titrate inotropes to off if not affecting hemodynamic status of patients and aggressively correct electrolyte imbalance. In the early period of our study we used digoxin before the administration of amiodarone. Some authors [14] didn’t recommend the use of digoxin in JET because its direct action of increasing excitability of all forms of myocardium, however, it was used to delay atrioventricular node bundle conduction maintaining a reasonable ventricular rate and to counteract the negative inotropic effect of Class III antiarrhythmic (sotalol and amiodarone). Recently amiodarone has gained popularity in JET treatment or prophylaxis. [5, 8] Many authors stated that class III antiarrhythmic drugs (sotalol and amiodarone) when given orally or intravenous, were shown to be almost devoid of negative inotropic effects [14, 18], however amiodarone use was associated with prolonged ICU stay in our JET patients.

In our series the median duration of JET was 30.5 h and many cases resolved smoothly without any hemodynamic instability with recurrence of a second episode of JET in 5.2% of the patients. Complications in the form of heart block requiring pacemaker insertion was encountered in 4 patients of the JET group. Junctional ectopic tachycardia significantly increased ICU stay but not the duration of the mechanical ventilation of hospital stay. This is explained by the benign course of our JET patients and the additional treatment required lead to increased ICU stay while there was no effect on total hospital stay. No difference in patients’ characteristics or the outcome of JET in patients who had JET onset intraoperatively versus those who had JET onset in the ICU. This can be explained by the overlap of the time frame between both groups and it is recommended for future to classify JET into early and late onset based on time of onset rather than the place of onset. Moreover, the duration of JET had an impact on the patients’ outcome since longer JET episodes lead to prolonged ICU and hospital stay which is expected due to the increase in time required for treating those patients compared with shorter JET episodes.

In summary, JET post tetralogy of Fallot repair can be predicted based on the preoperative variables. Preoperative cyanotic spells were associated with postoperative JET and preoperative B-blockers significantly reduced postoperative JET. Postoperative electrolytes imbalance played a role in JET pathogenesis. JET had a benign course and didn’t increase hospital mortality but it prolonged ICU stay. The outcome of JET was affected by the duration of JET episode but not the time of onset.

### Study strength and limitations

The major limitation of the study is the retrospective design with its inherited biases. However, this is an acceptable design for rare outcomes. Many of the limitations of previously published reports were managed in our study including limiting selection to a specific age and pathology in contrast to other studies which included all types of congenital heart disease and wide range of age. The relatively large number in our series and the high incidence of JET in this subset of patients properly powered the multivariable analysis. Missing of some variables is another limitation however missing values didn’t exceed 8% (1–7.1%) in the variables used in the analysis and multiple imputation is a suitable method to handle these missing variables. Recent studies [7, 9] showed that Dexmedetomidine has a role as a prophylactic and therapeutic agent for postoperative JET, however we didn’t use it in our patients which could be another limitation of the study.

## Conclusion

Junctional ectopic tachycardia is a frequent complication after Tetralogy of Fallot repair. It has a benign course; however, it prolongs ICU stay after TOF repair. Preoperative B-blockers reduced postoperative JET and patients with preoperative risk factors could benefit from preoperative arrhythmia prophylaxis. JET can be prevented by aggressive management of postoperative electrolyte disturbance. The outcome of JET was not affected by time of JET onset however, prolonged duration of JET had a negative impact of ICU and hospital stay.

## Abbreviations

BSA: Body surface area
CPB: Cardiopulmonary bypass
ICU: Intensive care unit
JET: Junctional Ectopic Tachycardia
RVOT: Right ventricular outflow tract
TAP: Trans-annular patch
TOF: Tetralogy of Fallot
